# Duck Egg-Drop Syndrome Caused by BYD Virus, a New Tembusu-Related Flavivirus

**DOI:** 10.1371/journal.pone.0018106

**Published:** 2011-03-24

**Authors:** Jingliang Su, Shuang Li, Xudong Hu, Xiuling Yu, Yongyue Wang, Peipei Liu, Xishan Lu, Guozhong Zhang, Xueying Hu, Di Liu, Xiaoxia Li, Wenliang Su, Hao Lu, Ngai Shing Mok, Peiyi Wang, Ming Wang, Kegong Tian, George F. Gao

**Affiliations:** 1 Key Laboratory of Zoonosis of Ministry of Agriculture, College of Veterinary Medicine, China Agricultural University, Beijing, China; 2 CAS Key Laboratory of Pathogenic Microbiology and Immunology, Institute of Microbiology, Chinese Academy of Sciences, Beijing, China; 3 China Animal Disease Control Center, Beijing, China; 4 Graduate University, Chinese Academy of Sciences, Beijing, China; 5 College of Veterinary Medicine, Huazhong Agricultural University, Wuhan, China; 6 Network Information Center, Institute of Microbiology, Chinese Academy of Sciences, Beijing, China; 7 Beijing Institutes of Life Sciences, Chinese Academy of Sciences, Beijing, China; University of Kansas Medical Center, United States of America

## Abstract

Since April 2010, a severe outbreak of duck viral infection, with egg drop, feed uptake decline and ovary-oviduct disease, has spread around the major duck-producing regions in China. A new virus, named BYD virus, was isolated in different areas, and a similar disease was reproduced in healthy egg-producing ducks, infecting with the isolated virus. The virus was re-isolated from the affected ducks and replicated well in primary duck embryo fibroblasts and Vero cells, causing the cytopathic effect. The virus was identified as an enveloped positive-stranded RNA virus with a size of approximately 55 nm in diameter. Genomic sequencing of the isolated virus revealed that it is closely related to Tembusu virus (a mosquito-borne Ntaya group flavivirus), with 87–91% nucleotide identity of the partial E (envelope) proteins to that of Tembusu virus and 72% of the entire genome coding sequence with Bagaza virus, the most closely related flavivirus with an entirely sequenced genome. Collectively our systematic studies fulfill Koch's postulates, and therefore, the causative agent of the duck egg drop syndrome occurring in China is a new flavivirus. Flavivirus is an emerging and re-emerging zoonotic pathogen and BYD virus that causes severe egg-drop, could be disastrous for the duck industry. More importantly its public health concerns should also be evaluated, and its epidemiology should be closely watched due to the zoonotic nature of flaviviruses.

## Introduction

Duck farming is a traditional agro-business in China and Southeast Asia, and Peking roast duck is well known worldwide. Several infectious pathogens affect the duck industry, such as duck hepatitis virus, duck enteritis virus, *Rimerella anatipestifer*, and *Escherichia coli* etc. [1]. To date, however, there is no report on duck infection by any flaviviruses with severe outcomes.

Flaviviruses are single-stranded positive-sense RNA viruses classified in the Genus *Flavivirus*, Family *Flaviviridae* with over 70 serotype members [2], of which several important vector-borne viruses (especially those with a zoonotic nature, e.g., yellow fever virus, West Nile virus, Japanese encephalitis virus, and tick-borne encephalitis virus) are members. They result in symptoms from mild febrile disease, encephalitis, hemorrhagic fever, and shock syndrome to death in both humans and animals [3]–[5]. Most flaviviruses are transmitted by hematophagous arthropod vectors, including mosquitoes and ticks [6], [7], and some flaviviruses have been isolated from these vectors that do not cause any known diseases to either animals or humans [8]. The flavivirus genome, approximately 10.5 kb in size, encodes three structural proteins [capsid (C), membrane (PrM and M) and envelope (E)] and seven non-structural proteins (NS1, NS2A, NS2B, NS3, NS4A, NS4B and NS5) in one open reading frame with subsequent cleavage [9]. Among these proteins, E protein plays an important role in virus receptor-binding, entry and fusion.

Starting in April 2010, a severe viral disease spread around the duck-producing regions in China, even in autumn when there is low or no mosquito activity in northern China. The affected ducks (including Pekin ducks, Muscovy ducks and domesticated mallards or Ma Ya) manifest a clinical symptom with heavy egg drop. On some duck farms, the disease was devastating, completely eliminating successful duck reproduction. Therefore this viral disease has caused a serious economic loss. We performed a systematic investigation, from epidemiology, pathogen isolation, virus characterization, disease reproduction by infection with the isolated virus (fulfilling Koch's postulates), to virus genome sequencing and found that the duck egg drop disease was caused by a new flavivirus, BYD virus, that is closely related to Tembusu virus.

## Results

### Epidemiological and clinical features

**Figure 1:**
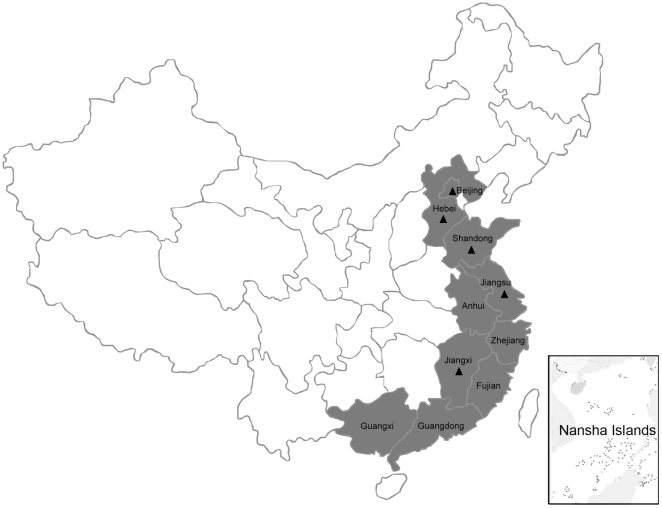
Regions of BYDV infection outbreaks in China. The provinces or autonomous cities (regions) affected are indicated in gray. Regions from which viruses were isolated and comfirmed by RT-PCR/sequencing are labeled with triangles.

**Figure 2:**
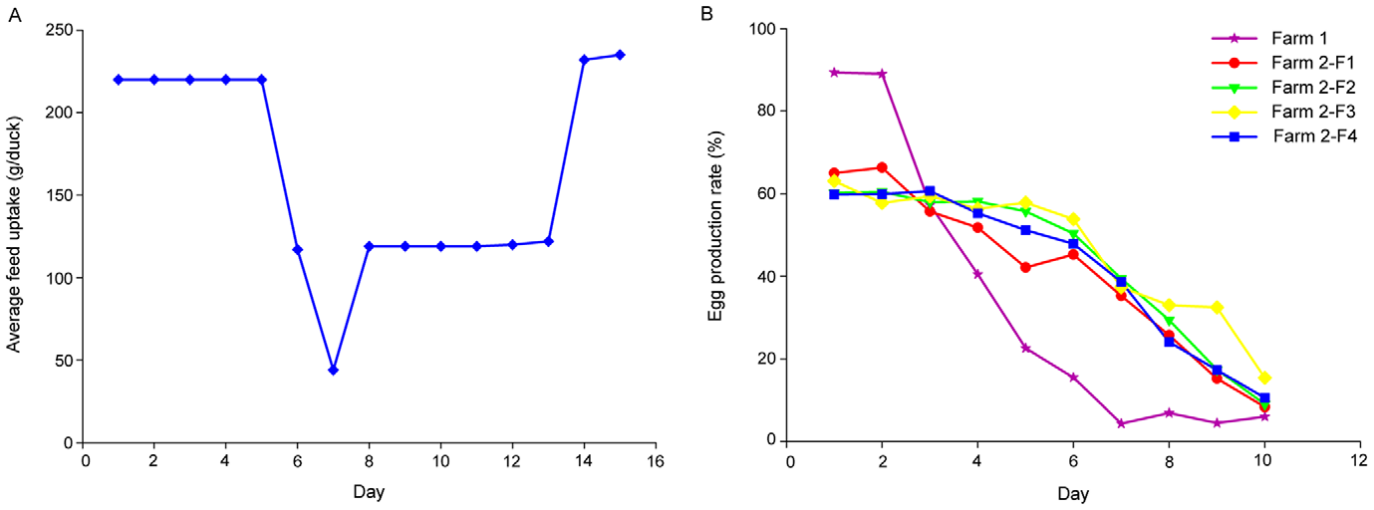
The course of natural disease. Representative of the average feed uptake of the infected flock at Farm 1 (A); Daily egg production rate represented by two infected duck farms (B). The flocks at Farm 1 were 35 weeks old, and Farm 2 flocks 1–4 (Farm 2-F1, -F2, F3, -F4) are four different flocks (76 weeks old) at another representative farm.

In April 2010, an egg drop syndrome of unknown etiology was found in several duck farms in Southeast China. Based on our preliminary epidemiological data, the disease quickly spread to most of the duck-producing regions in China including many of the coastal provinces and neighboring regions, Anhui Province, Beijing Autonomous City, Hebei Province, Fujian Province, Guangdong Province, Guangxi Province, Jiangsu Province, Jiangxi Province, Shandong Province and Zhejiang Province (Fig. 1). The disease affected both meat-type duck breeder flocks and egg-laying duck strains. Because duck farming in China is still practiced in a traditional way, it was difficult to determine an exact number of affected ducks. However, from our limited epidemiological statistics, using Fujian, Shandong and Zhejiang Provinces as examples, the affected ducks total at least 810,000; 1,500,000 and 2,100,000 (respectively) in these provinces. Typical affected duck flocks are shown in Figure 2. The infected flocks are characterized by a sudden decline of feed uptake (Fig. 2A) accompanied by a heavy drop in egg production (Fig. 2B). Indeed, the egg production rate dropped severely to ≤10% within 5 days. Greenish diarrhea was also frequently observed in the flocks. As the disease progressed, some ducks exhibited an uncordinated gait, and were reluctant or unable to walk. The total mortality ranged from 5 to 15% depending on the management conditions of the infected flocks.

### Pathology

**Figure 3:**
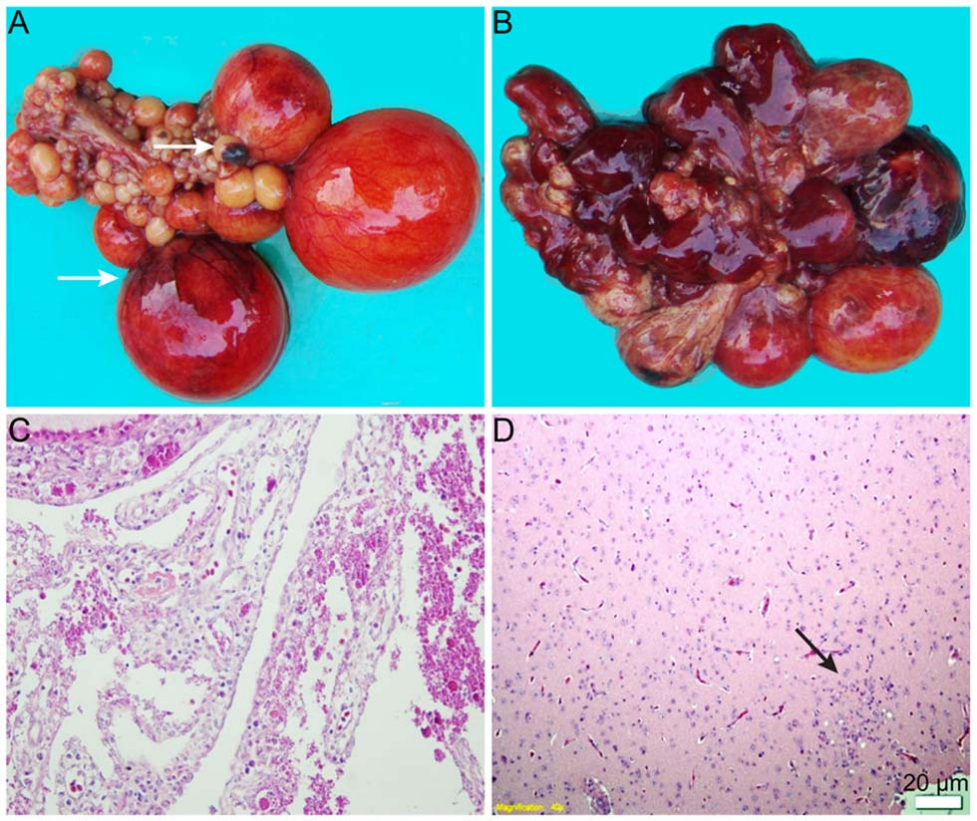
Gross lesions and histopathology of the clinical samples. Mild hemorrhage of ovarian follicles (arrow) in the early stage of infection (A); Severe hamorrhage and regression of ovarian follicles (B); HE stained ovary section, the follicles ruptured and filled with round or granular eosinophilic bodies (C). HE-stained brain section showing focal gliosis (arrow) (D).

At necropsy, severe ovarian hemorrhage, ovaritis and regression were consistently observed in the affected ducks (Fig. 3A and B). Ruptured ovarian follicles and peritonitis were also found in some affected ducks. Enlarged spleen was occasionally found.

Histopathologically, the most obvious features of the disease were ovarian hemorrhage, follicle atresia and rupture. The ruptured follicles and interstitial tissues were filled with round or granular eosinophilic bodies (Fig. 3C). Similar eosinophilic bodies were also observed in the serous side of multiple visceral organs. Focal gliosis occurred occasionally in the brain, with lymphocyte infiltration under cranial arachnoid (Fig. 3D).

### Isolation and characterization of the BYD virus

**Figure 4:**
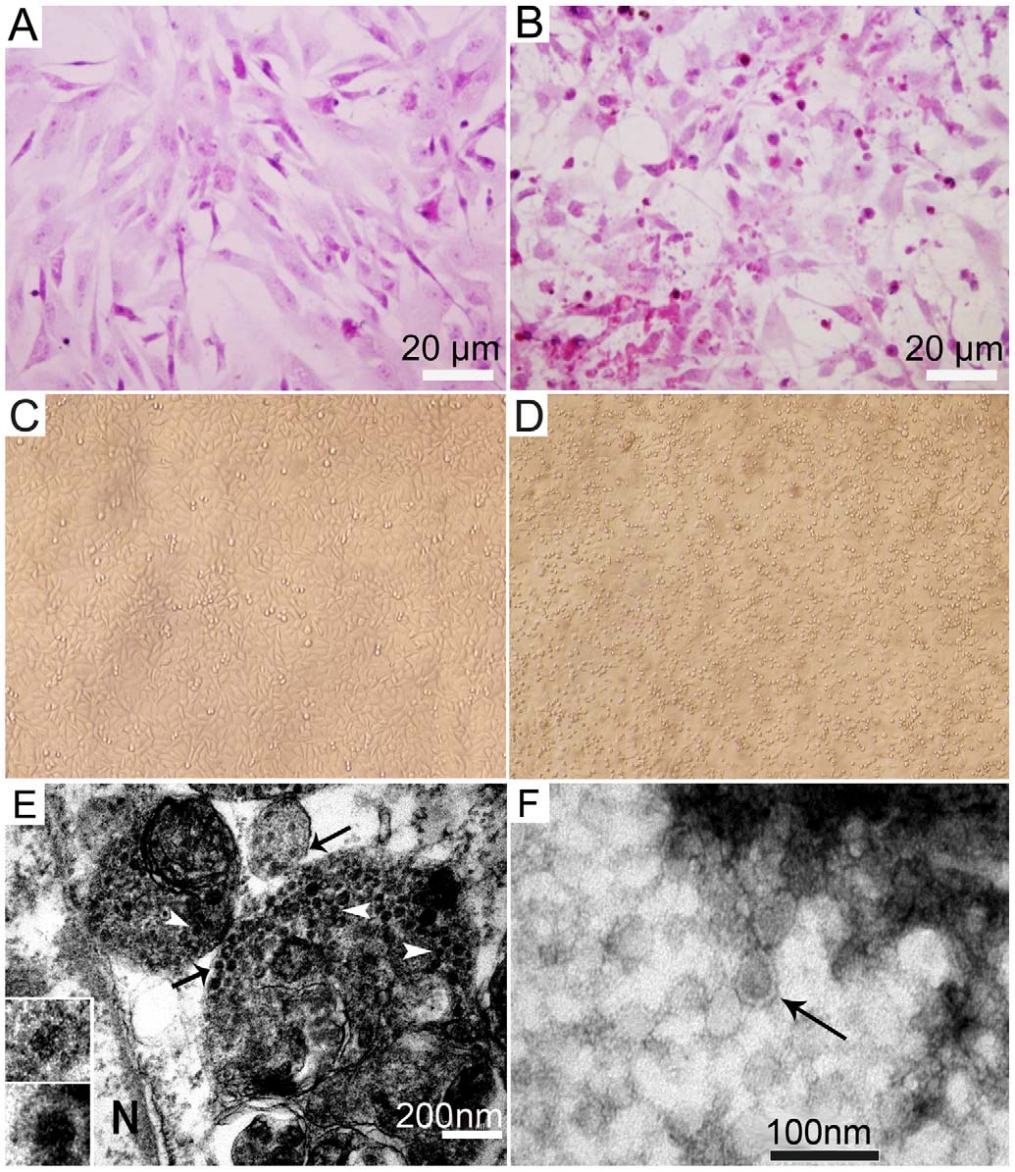
CPE in BYDV infected DEFs and electron micrographs of virus particles. HE-stained, non-infected DEF control (A); HE stained infected cells, 52 hrs post-infection, showing cell disruption with a large number of red-stained particles (B); Non-infected Vero cell monolayer (C); Infected Vero cells rounded up and focal detachment 60 hrs post-infection (D); Thin section of the infected cell, 24 hrs post-infection, showing dense particles (white arrow head) and virions (arrow) within the vesicles in the cytoplasm (N, nucleus). Insets are magnified virions (E); Negative staining of the purified BYDV from the cell culture supernatants (arrow indicates the typical enveloped flavivirus) (F).

Conventional infections with known bacteria or viruses were ruled out from our bacteria/virus isolations, especially. the possible influenza A virus and EDS-76 adenovirus. Thus, we next attempted to isolate any new pathogens. Brain and ovary samples from affected ducks were processed for virus isolation using embryonated duck eggs and embryos died 3–4 days post-inoculation. The allantoic fluids were then harvested for passaging. At the fifth passage, the allantoic fluid had an infectivity titer of 10^5.8^ELD_50_/ml(i.e., the 50% embyro infective dose). The virus produced a marked cytopathic effect (CPE) in the primary duck embryo fibroblasts (DEFs) 36 hrs after inoculation. The infected cells ruptured, and eosinophilic particles were found with hematoxylin and eosin (HE) staining (Fig. 4A and B). The virus also replicated in Vero cells, and focal CPE appeared with the cells rounding up and floating free from the surface of the flask 60 hrs after infection (Fig. 4C and D). The isolated virus was named as Baiyangdian (BYD) virus (BYDV) after the region name where the virus was first isolated. We isolated a dozen of the BYDV strains (four from Beijing, two from Hebei, three from Shandong, one from Jiangsu and two from Jiangxi, respectively) from the affected duck brain and ovary tissues in total but focused on a single strain (BYDV-byd1) for the following studies, including genomic sequencing.

When treated with chloroform, the infectivity of the BYDV droped from 10^5.8.^ to <10 ELD_50_/ml, indicating that it was an enveloped virus. To determine the nucleic acid type, the virus was assayed in DEF cultures containing 5-bromo-2′-deoxyuridine (BrdU). The infectious titers of the BYDV-byd1, duck reovirus, and pseudorabies virus (RNA and DNA virus controls) in the presence and absence of BrdU were 2×10^6.4^ and 2×10^6.2^, 2×10^5.4^ and 2×10^5.6^, <10 and 2×10^4^ TCID_50_/ml, respectively (Table 1). These results suggest that BYDV is an RNA virus, ruling out the possibilty that the disease was caused by the well-known DNA virus, EDS-76 adenovirus,which causes egg drop in poultry, including ducks [10].

**Table 1:** Results of BrdU inhibitory assay in DEF culture.

Virus	Titers in DEF culture with BrdU- supplemented medium(TCID_50_/ml)	Titers in DEF culture withplain medium(TCID_50_/ml)
BYD virus	2×10^6.4^	2×10^6.2^
Duck reovirus	2×10^5.4^	2×10^5.6^
Pseudorabies virus	2×10^4.0^	<10

In an ultrathin section of infected DEF cells obtained 24 hrs post-infection, dense particles measuring 30 to 40 nm in diameter were found within cytoplasmic vesicles in large numbers (Fig. 4E) by electron microscopy (EM). Virions near the vesicle membrane measured approximately 55 nm in diameter, and exhibited a dense core 30 to 40 nm in diameter. Similar morphological observations were described for Dengue-2 virus [11]. The enveloped viral particles were also observed in negatively stained samples prepared from the concentrated cell culture supernatants and allantoic fluids (Fig. 4F).

We used the ELISA method for BYDV-specific antibody detection in the convalescent sera. The optical density at 450 nm (OD_450)_ of sera from the recovered ducks (n = 20) were 3-fold higher than the control group (n = 10). This further confirmed the BYDV infection in the duck population.

### Reproduction of the egg drop disease with experimental BYDV infection

**Figure 5:**
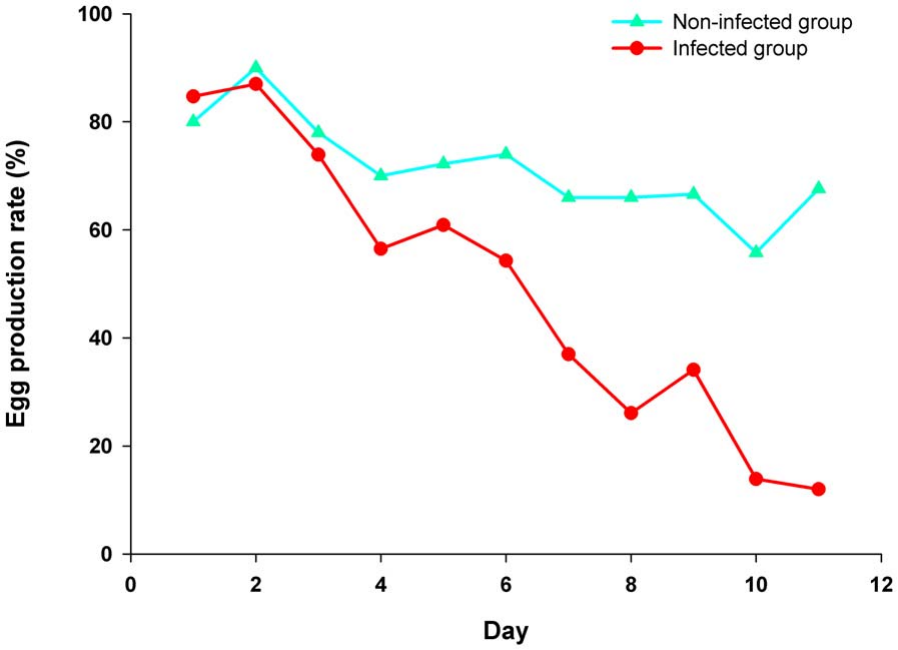
Daily egg production rate before and after experimental infection. The decline of egg production rate was evident, similar to the natural infection.

**Figure 6:**
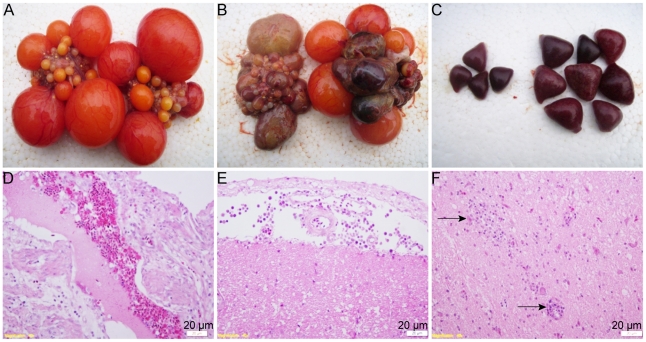
Pathological changes of the experimentally-infected ducks. Mock-infected control (A); Severe hemorrhage and regression of ovarian follicles (B); Spleens from mock-infected control (left) and enlarged spleens from the infected ducks (C); HE-stained ovary section showing hemorrhage and follicle rupture with red stained bodies (D); HE-stained brain section revealing lymphocytes and mononuclear cell infiltration under cranial arachnoid (E) and focal gliosis (arrow) (F).

To confirm the etiological agent of this outbreak, it was essential to reproduce the disease by experimental infection of laying ducks with BYDV-byd1 strain. Thus, ducks with a high egg production rate were challenged with BYDV. As shown in Figure 5, the infected group experienced a significant egg drop from 60.9% to 12% during a 6-day infection (Fig. 5). Necropsies revealed severe ovary hemorrhage and regression similar to the clinical cases on days 3, 4, 5, and 6 post-infection (Fig. 6A and B). Enlarged spleen was also observed at necropsy (Fig. 6C) and peritonitis was evident in most infected ducks. Tissue sections of the ovary and brain mimicked those in the naturally infected ducks (Fig. 6D–F). The virus was also successfully recovered from the infected ducks and confirmed by reverse transcription (RT)-PCR and genomic sequencing in subsequent experiments.

### Genomic sequencing

**Figure 7:**
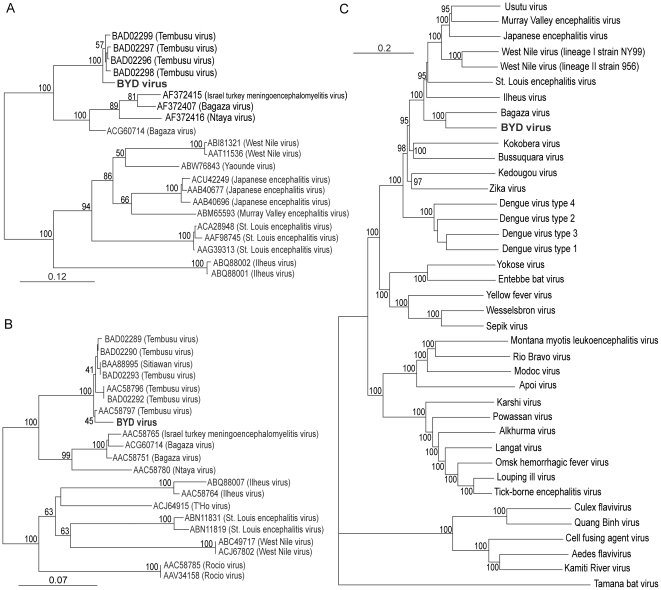
Phylogenetic relationships of the isolated BYDV with other flaviviruses. Nucleotide sequence comparisons of the E protein (A), NS5 protein (B) and the genome coding polyprotein (C). BYDV is labeled in bold.

The sensitivity to chloroform treatment and the resistance to DNA virus inhibitors indicated that BYDV might be an enveloped RNA virus. Because an egg-production drop is also observed in avian flocks during eastern equine encephalitis virus (EEEV) infection [12], we chose a pair of primers specific for EEEV for RT-PCR. This primer set yields a 433-bp product in EEEV [13], but a 629-bp fragment was amplified from BYDV-byd1 after RT-PCR. Sequencing and BLAST analysis recealed a 87–91%/87% nucleotide identity with a partial coding sequence from the Tembusu/Sitiawan virus E gene (encoding the envelope protein) (Fig. 7A). A partial NS5 gene nucleotide sequence also indicates that BYDV is closely related to Tembusu virus or Sitiawan virus, with_88–92% and 88% identity,respectively (Fig. 7B). Due to the lack of a complete genome sequence for the Tembusu and Sitiawan viruses, we designed pairs of overlapping primers according to the conserved region in 10 out of 38 available flavivirus complete genome sequences from GenBank. After assembling the sequences of all RT-PCR products, we obtained the entire coding sequence of BYDV-byd1 (GenBank Accession JF312912). A phylogenetic tree of the polyprotein genes indicates that BYDV is closely related (72% identity) to Bagaza virus (Fig. 7C), an African flavivirus isolate that causes diseases in both humans and animals [4], [14], Thus, all of these data demonstrate that BYDV is a new flavivirus, which is most-closely related to the Tembusu and Sitiawan viruses.

**Figure 8:**
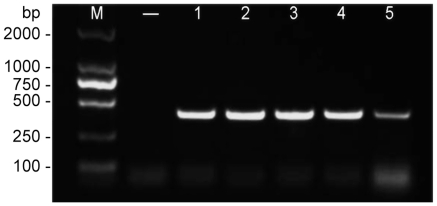
Detection of the viral RNA from clinically infected duck samples. Infection in duck brain tissues collected from different regions was assessed using RT-PCR with BYDV E gene-specific primers. -: negative control; 1,2,3,4 and 5: samples from Hebei, Jiangxi, Jiangsu, Beijing and Shandong provinces. These PCR products were sequenced and their identities were confirmed.

After the BYDV genomic sequencing, partial E genes (401-bp product) amplified from the suspected brain tissues of the affected ducks were used for clinical diagnoses and virus screening by RT-PCR using specific primer pairs. Representative results of samples from five different provinces are shown in Figure 8. All of the PCR products were subjected to sequencing for confirmation.

## Discussion

The sudden outbreak and quick spread of an egg drop syndrome in the major duck-producing regions in China has resulted in serious economic loss. This prompted us to conduct a thorough investigation of the cause of the disease. From preliminary epidemiology, pathogen isolation, and reproduction of the clinical disease by the isolated virus, BYDV, Koch's postulates were fulfilled, and a new flavivirus causing a serious duck disease was identified. This is the first severe duck disease caused by a zoonotic flavivirus ever reported. It had a very significant effect on the duck industry, especially in Southeast Asia where duck farming is most popular and mosquitoes are active. Although the transmission vector of the BYDV was not defined in our study, most (if not all) of its closely related viruses are transmitted by mosquitoes or ticks. This should be addressed in the future studies (e.g., the isolation of BYDV from mosquitoes should be attempted). However, it should be noted that the virus infection in egg-laying ducks in this outbreak continued into autumn when mosquito activity is low in northern China.

Flaviviruses are notorious for their severe infective and zoonotic nature. Indeed, they can cause serious diseases for both humans and animals. Infections of poultry by flaviviruses are not uncommon, such as West Nile virus [15], [16] and Israel turkey meningoencephalitis virus in geese and turkeys [17]. Further, a newly emerging African flavivirus, Bagaza virus [18], both causes human illness [4], [19] and infects birds in Africa [20], [21]. However this is the first report of flavivirus infection in ducks with a severe outcome and the future implications of such findings should be heeded. It is noteworthy that, in our initial virus gene amplification screening, a hypothesis for PCR primer design, based on an early report that EEEV causes egg-drop in turkey [12], helped us have obtained BYDV gene fragments. Our findings revealed that the BYDV is closely related to Tembusu virus. Tembusu virus was initially isolated from mosquitoes as early as 1955, but its relevance to human or animal health has never been fully established. However, both the existence of antibodies in humans against Tembusu virus [22]–[26] and a new closely-related virus (Sitiawan virus [27]) being correlated to chick encephalitis and retarded growth, raise serious concerns about BYDV as an emerging pathogen. In any event, the relatedness of Tembusu virus, Sitiawan virus and BYDV requires further investigation.

The public health significance of BYDV isolation as a duck pathogen can not be underevalud. Potential infection or asymptomatic infection of humans must be evaluated as soon as possible because duck consumption in Asia, especially in China, is extremely heavy. Close contact between humans and ducks or duck products is inevitable. Therefore, for both potential public health concerns and the duck industry, vaccine development against BYDV should be considered. Indeed, several succussful vaccines for flaviviruses have been developed and widely used (e.g., yellow fever virus and Japanese encephalitis virus), so a vaccine for BYDV may be feasible.

With frequent isolations, flaviviruses continue to threaten human health and animal industries. Expansion of dengue virus, West Nile virus, and Kyasanur forest virus to the northern hemisphere, as well as the emergnce of new viruses (e.g., such as BYDV, Sitiawan virus [27], and Bagaza virus [18]) and re-emergence of yellow fever virus and Japanese encephalitis virus, all indicate that flaviviruses can not be neglected. Future surveillence should be enhanced worldwide.

## Materials and Methods

### Clinical and pathological investigations

We were notified by the local veterinary laboratories and duck farmers of an unknown egg-drop disease. Clinical examination and the production records were checked for several affected flocks in different areas. To exclude avian influenza virus infection, serum samples were collected from the affected flocks, and antibody titers against H5 subtype avian influenza A virus were tested with hemagglutination inhibition (HI) assays according to the standard method (http://www.oie.int/eng/normes/mmanual/2008/pdf/2.03.04_AI.pdf). Ducks in the affected flocks showing morbidity or death within 6 hrs were taken to our laboratory for necropsy. Tissue samples were fixed in 10% neutral buffered formalin solution for histopathology. Simultaneously, brain, ovary and spleen tissues were frozen at −80°C for further analysis. The fixed tissues were routinely processed and embedded in paraffin. Sections (4 µm thick) were prepared from the paraffin blocks and stained with HE.

### Virus isolation

Brain or ovary tissues from the affected ducks were homogenized in sterile phosphate-buffered saline (PBS, pH 7.2) to give a 20% suspension (w/v). After centrifugation at 3,000 g for 20 min, the supernatants were filtered through a 0.2-µm syringe-driven filter. The filtered suspension was then inoculated into five 10-day-old Pekin duck embryonated eggs (0.3 ml/embryo) via the allantoic sac. Embryos were checked daily, and the amnio-allantoic fluids of the infected embryos were collected for further passage. A suspected virus was isolated and it was named BYDV (The strain for further study in this report is named BYDV-byd1).

For cell culture passage, DEFs were prepared from 12-day-old duck embryos according to standard protocols, and Vero cells were grown in Dulbecco's modified Eagle's medium (DMEM; Invitrogen) supplemented with 10% fetal calf serum (FCS), 100 IU/ml penicillin and 100 mg/ml streptomycin. Cell monolayers were infected with 10-fold diluted allantoic fluid from the first passage of the duck embryos. The cultures were incubated at 37°C with 5% CO_2_ and checked daily for CPE. When 75% of the cells in culture demonstrated CPE, the cultures were harvested to infect fresh cells.

Allantoic fluid and the cell culture supernatants from the first five passages were tested for hemagglutinating activity with a 1% suspension of chicken erythrocytes to exclude avian hemagglutinating viruses (e.g., influenza virus and EDS-76 virus).

### Virus characterization

To determine whether the isolated virus had a lipid envelope, the allantoic fluid virus (fifth passage) was treated with 5% (v/v) chloroform and the infectivity was assayed by duck embryo inoculation as descried previously [28]. For nucleic acid type determination, virus replication was tested in DEF cultures with or without the presence of BrdU in the medium. Duck reovirus and pseudorabies virus were used as controls.

### ELISA for antibody detection

To detect an antibody response to the BYD virus in infected ducks, an ELISA was developed based on the method described by Hlinak et al. [29]. Briefly, BYD virus was propagated in DEF cultures. The DEF cultures were then harvested and subjected to freezing and thawing three times. Cell debris was removed from culture supernatant by centrifugation at 12,500 g for 45 min at 4°C, and the virus particles were concentrated by ultracentrifugation at 45,000 r.p.m in a BECKMAN 70Ti rotor for 3 hrs. The virus pellet was resuspended in a small volume of sterile PBS (pH 7.2). Further purification was conducted by discontinuous gradient centrifugation with 20% and 50% sucrose (w/w). Virus particles recovered from the interface between the two sucrose layers were used as coating antigen. Microtiter plates were coated with 100 µl/well BYD virus antigen at a concentration of 6 µg/ml in carbonate/bicarbonate buffer (pH 9.6) overnight at 4°C. Plates were washed three times with PBS (pH 7.4) containing 0.05% Tween 20 (PBS-T) and treated with blocking buffer (5% skim milk) at 37°C for 2 hrs. After washing, 100 µl of duck sera (diluted 1∶200 in PBS-T) was added to each well and incubated at 37°C for 1 hr. After incubation, plates were washed three times, and 100 µl/well peroxidase-labeled goat anti duck IgG (H+L) (KPL, USA) was added at the working dilution of 1∶500. Following this, the wells were washed and 100 µl/well tetramethylbenzidine (TMB) substrate solution was added. The reaction was incubated for 15 min at room temperature and stopped with 2 M H_2_SO_4_. The OD_450_ in each well was measured using an ELISA reader (Thermo Scientific Multiskan MK3). Twenty of serum samples from ducks recovered from outbreaks were tested, and ten of serum samples from normal ducks were used as control. The results are reported as the OD_450_ of test well/OD_450_ of the control well.

### EM

For EM, virus-infected and control DEF cells were fixed with 2.5% glutaraldehyde at 24, 36 and 48 hrs post-infection. Ultrathin sections were processed and stained with uranyl acetate and lead citrate. The samples were then examined with a Hitachi TEM H-7500 as described previously [11]. Additionally, the DEF cultures were harvested and concentrated by ultracentrifugation as above. The pellet was resuspended to 1/100 of the original volume in PBS and examined by negative contrast EM (Hitachi TEM H-7500).

### Experimental reproduction of the disease yia infection with the isolated virus

To evaluate the pathogenicity of the BYDV, experimental infection was conducted in 96 440-day-old healthy laying ducks (no SPF ducks were available). Ducks were transferred to our animal facility and adapted to the new environment for 5 days to minimize the effect of shipping stress. Forty-six ducks were simultaneously infected by intramuscular and intranasal injection with 0.6 ml and 0.4 ml of the BYDV-byd1 virus (10^5.8^ ELD_50_/ml), respectively. Additionally, fifty ducks were mock-infected with sterile PBS in the same manner. Ducks were monitored daily for egg production and killed on days 3, 4, 5 and 6 post-infection. Tissues were collected for pathological examination and virus isolation. DEF cultures were used to recover the virus from brain and ovary samples. The recovered virus was confirmed by RT-PCR and subsequent sequencing.

### Ethics Statement

All of the animal infection experiments were conducted in accordance with the guidelines of Beijing Municipality on the Review of Welfare and Ethics of Laboratory Animals approved by the Beijing Municipality Administration Office of Laboratory Animals (BAOLA). Animal infection experiments were conducted under the protolcol (CAU-AEC-2010-0603) approved by the China Agricultural University Animal Ethics Committee.

### Virus gene amplification and genome sequencing

Ultracentrifuge-purified BYDV-byd1 virus (fifth passage on DEF monolayer) was used for virus genome amplification. Because all of the aforementioned measures indicated that BYDV was an RNA virus, viral RNAs were extracted using a QIAamp Viral RNA Mini kit (Qiagen) following the manufacturer's protocol. cDNA was synthesized using the SuperScript III First-Strand Synthesis System for RT-PCR (Invitrogen). Initially we attempted to randomly amplify viral gene fragments by the random priming method [30] but failed to recover anything. Based on the fact that the EEEV causes egg-drop syndrome in turkeys [12], we hypothesized that BYDV might be closely related to it. Therefore, primers designed to amplify EEEV (EEEVF5640 and EEEVR6072) were used as reported (Table 2) [13]. The amplified DNA products were analyzed on a 1.0% agarose gel, and pieces of gel containing the amplified DNA bands were excised and purified using a gel extraction kit (Takara Bio). Interestingly we recovered an approximately 600-bp fragment using these primers. After purification, the PCR products were sequenced by Beijing Sunbiotech Co., Ltd. BLAST analysis revealed that the sequence of the ∼600-bp fragment is closely related to flaviviruses but not alphaviruses. Based on the sequence of this fragment and 10 phylogenetically closely related flavivirus complete genome sequences, the primer sets AF(f)/CR(r), CF(f)/ER(r), DF(f)/GR(r), GF(f)/N6R(r), N6F(f)/LR(r) and 12F(f)/13R(r) used for genomic sequencing were designed referred to the sequence of Bagaza virus/NC_012534 based on the conserved region of the 10 flavivirused using BioEdit (version 7.0.5.3) and MEGA (version 3.1) (Table 2). Using these primers, fragments of approximately 1200-bp, 1000-bp, 1500-bp, 1300-bp, 4300-bp and 1800-bp were obtained. Primers CT-C, CT-G, CT-N6, and CT-L were designed based on sequence results to eliminate base error introduced by the first set of primers. All sequencing data from the PCR products were assembled using Vector NTI (Invitrogen), compiled and edited by using Bioedit (Version 7.0.5.3). Bootstrapped (1000 times) neighbor-joining phylogenetic trees were generated by MEGA (version 3.1). A new primer pair designed from the sequenced E gene TV-3(f) and TV-3(r) was used to detect viral genes in clinical samples collected from different affected regions in China.

**Table 2:** Primers and viruses referred to in this study.

Name	Sequence (5′→3)	Genomic position	Virus & GenBank accession number	Remark
EEEV5640	CGGCAGCGGAATTTGACGAG	5640–5659	Eastern equine encephalitis virus/NC_AY705240	[13]
EEEV6072	ACTTTGACGGCCACTTCTGCTGATGA	6047–6072	Eastern equine encephalitis virus/NC_AY705240	[13]
AF(f) *	AGAAGTTCATCTGTGTGA	1–18	Bagaza virus/NC_012534	Newly designed
CR(r)†	CCCTTTCCGAACAGTCCACA	1274–1293	Bagaza virus/NC_012534	Newly designed
CF(f)	TGTGGACTGTTCGGAAAGGG	1274–1293	Bagaza virus/NC_012534	Newly designed
ER(r)	GATCCAAAGTCCCATGC	2234–2250	Bagaza virus/NC_012534	Newly designed
DF(f)	GACACAGGGCATGGGAC	1910–1926	Bagaza virus/NC_012534	Newly designed
GR(r)	TCCATTCCATACCAGCA	3449–3465	Bagaza virus/NC_012534	Newly designed
GF(f)	TGCTGGTATGGAATGGA	3449–3465	Bagaza virus/NC_012534	Newly designed
N6R(r)	ACATGCCACATCGTGTGGAA	4730–4749	Bagaza virus/NC_012534	Newly designed
N6F(f)	TTCCACACGATGTGGCATGT	4730–4749	Bagaza virus/NC_012534	Newly designed
LR(r)	TTTCCCATCATGTTGTA	9017–9033	Bagaza virus/NC_012534	Newly designed
12F(f) §	TAYAACATGATGGGVAA	9017–9033‡	-	Newly designed
13R(r) §	GGGTCTCCWCTAACCTCTAGTCCKT	10784–10808‡	-	Newly designed
CT-C	AGTGGATCGATGTCGTTCT	917–935	BYD virus/JF312912	Newly designed
CT-G	GAGTGAACTCATCATACC	3078–3095	BYD virus/JF312912	Newly designed
CT-N6	CAGGTTACTGGATGACAACC	4460–4479	BYD virus/JF312912	Newly designed
CT-L	GATGTGGAACTTTGTTGGC	8706–8724	BYD virus/JF312912	Newly designed
TV-3(f)	GCCACGGAATTAGCGGTTGT	1009–1028	BYD virus/JF312912	Newly designed
TV-3(r)	TAATCCTCCATCTCAGCGGTGTAG	1386–1409	BYD virus/JF312912	Newly designed

*f, forward primer.

†r, reverse primer.

‡Genomic position of degenerate primer in Bagaza virus. NC_012534.

§Degenerate primers were designed according to the conserved region in 10 flavivirus complete genome sequences from GenBank, The virus name and GenBank accession numbers are Usutu virus NC_006551, Murray Valley encephalitis virus NC_000943, Japanese encephalitis virus NC_001437, West Nile virus (lineage I strain NY99) NC_009942, West Nile virus (lineage II strain 956) NC_001563, St.Louis encephalitis virus NC_007580, Ilheus virus NC_009028, Bagaza virus NC_012534, Kokobera virus NC_009029 and Bussuquara virus NC_009026.
